# Regulatory Role of MicroRNAs in Muscle Atrophy during Exercise Intervention

**DOI:** 10.3390/ijms19020405

**Published:** 2018-01-30

**Authors:** Shufang Zhang, Ning Chen

**Affiliations:** 1 Graduate School, Wuhan Sports University, Wuhan 430079, China; shufangzhang0308@163.com; 2 College of Sports Science and Technology, Wuhan Sports University, Wuhan 430205, China; 3 Tianjiu Research and Development Center for Exercise Nutrition and Foods, Hubei Key Laboratory of Sport Training and Monitoring, College of Health Science, Wuhan Sports University, Wuhan 430079, China

**Keywords:** microRNA, myogenesis, muscle atrophy, exercise, sport injury

## Abstract

Skeletal muscle comprising approximately 40% of body weight is highly important for locomotion and metabolic homeostasis. The growth and regeneration of skeletal muscle are highly organized processes; thus, it is not surprising to reveal certain complexity during these regulatory processes. Recently, a large number of evidence indicate that microRNAs can result in obvious impacts on growth, regeneration and metabolism of skeletal muscle. In this review, recent research achievements of microRNAs in regulating myogenesis, atrophy and aging during exercise intervention are discussed, which will provide the guidance for developing potential applications of microRNAs in health promotion and rehabilitation of sports injuries.

## 1. Introduction

Maintaining the function of skeletal muscle is the prerequisite of keeping individual health and independent living during the whole life cycle [1]. The body needs an effective pathway to regulate growth, regeneration and metabolism of skeletal muscle so as to make skeletal muscle at its best state [2]. Since a series of factors including neuromuscular disease, chronic disease and aging have an impact on the functions of skeletal muscle [3,4], it is important to define its influencing mechanism. The exploration of microRNAs (miRNAs) will play a positive role in broadening our understanding of controlling factors for skeletal muscle function, and improving the understanding and application of current therapeutic strategies in skeletal muscle diseases [5]. This article summarizes the latest research achievements of microRNAs associated with skeletal muscle function, explores the functions and corresponding regulatory mechanisms of miRNAs in some physiological and pathological conditions, in an attempt to provide the reference for developing novel and effective intervention strategies for maintaining and improving the functions of skeletal muscle.

## 2. Functional Mechanism and Classification of miRNAs

MiRNA, as an endogenous tiny RNA with the length of 20–24 nucleotides, is capable of horizontally regulating gene expression after being transcribed. Since the discovery of Lin4 miRNA in 1993, the growing amount of miRNAs and their corresponding targets have been screened and identified [6]. Current research has demonstrated that miRNAs have many significant regulatory effects in cells.

MiRNAs are presented in various forms, and the most primitive one is pri-mRNA, which is composed of 300–1000 base groups [7]. Affected by Dorsha Rnact, pri-mRNAs are cut into 70-basic-group pre-mRNAs. Then, the intra-nuclear pre-mRNA is transferred to the cytoplast under the action of the transporter Exportin5, and becomes a 21–25-base-group double-chain miRNA through the digestion of Dicer enzyme [8]. One of the miRNA chains is combined with RNA-induced RISC, forming a RISC compound that is then linked to mRNA. The incomplete complementary pairing of the flat-chain miRNA and its target 3′-UTR prevents gene translation and mRNA degradation. Each miRNA has various target genes, and several miRNAs can jointly regulate the same gene [9].

With the tissue variability, miRNAs in some tissues are highly or specifically expressed. Based on their expression levels in muscle tissues, miRNAs are divided into two types. The first type is called myomiR, which is only expressed in muscle tissues. The myomiR includes miR-1 and miR-133 expressed in both myocardium and skeletal muscle [10], and miR-206, miR-208, miR-208b, miR-486, miR-489 and miR-499 only expressed in skeletal muscle [11,12,13]. The second type of miRNA is expressed in both muscle and other tissues, such as miR-23, miR-181 and miR-24 [14]. These two types of miRNAs can regulate the proliferation and differentiation of skeletal muscle.

## 3. MiRNAs for Regulating the Development of Skeletal Muscle

The skeletal muscle fiber, belonging to the cell type that is terminally undifferentiated, mainly results from myoblast differentiation. The satellite cells in skeletal muscle are a kind of myoblasts in mature skeletal muscle tissue to form new muscle fibers or to mix with former muscle fibers for realizing the growth and development of skeletal muscle through proliferation and differentiation [15]. The myogenesis of skeletal muscle requires a lot of interworking factors, including cell withdrawing from their cell cycles, the expression of muscle-specific proteins, the integration of multinuclear myotubes, and the formation of contractible muscle fibers [16]. The growth and development processes of muscle are controlled by a series of critical transcription factors including helix-loop-helix (HLH), myogenic regulatory factor (MRF), myogenic factor 5 (Myf5), MRF4, myocyte enhancer factor 2 (MEF2), and serum response factor (SRF) [17,18]. Recent research has confirmed that miRNAs have played an important regulatory role in cell proliferation and differentiation, thus regulating the growth of skeletal muscle [19].

Currently, the studies on muscle-specific miR-1, miR-133 and miR-206 have gained tremendous attention to explore miRNA functions in myogenesis, and growing data from such research can provide the comprehensive explanation for the mechanisms of myogenesis and the defined signal pathways for cell proliferation and differentiation [10,17]. However, during the growth and development processes of skeletal muscle, the full effects of muscle-specific miRNAs and the functions of muscle non-specific miRNAs remain to be further explored.

The understanding of the functions of miR-1 and miR-133 is the first significant step for us to know the effects of miRNAs on regulating the growth and development of skeletal muscle. MiR-1 presents high expression in skeletal muscle and myocardium across species (from fruit flies to humans) [20]. After being transcribed by a common polycistron gene during muscle development process, miR-1 and miR-133 can regulate the growth and differentiation of muscle tissue via adjusting the activity of SRF and MEF2 [21]. Histone deacetylase 4 (HDAC4) serves as the transcription suppressor during muscle gene expression [22]. Recent investigations have confirmed that miR-1 can restrain HDAC4 to promote the growth of muscle, and it also can play a critical role in myoblast differentiation in the presence of MEF2. Therefore, the mechanism of miR-1 for promoting the differentiation of muscle fiber may reduce the expression of HDAC4 and strengthen the activity of MEF2 [23]. Similar to the function of miR-1, miR-206 can also result in the enhanced differentiation of myoblasts [24]. Gap junction protein connexin43 (Cx43) and Pola1 have been confirmed as the regulation targets of miR-206 [25]. Cx43 should be involved at the initial stage of myogenesis, and its expression is rapidly reduced after the induced differentiation. Hence, it is likely that miR-206 reduces mutual contact of muscle fibers through lowering Cx43 expression [25]. The adoption of miR-206 can lead to the decreased expression of Pola1, which will reduce DNA synthesis at the initial differentiation stage and restrain cell proliferation during the period of myotube formation [26]. Different from the functions of miR-1 and miR-206, miR-133 can promote myoblast proliferation via suppressing SRF and restraining myoblast differentiation [22,24]. Another muscle-enriched miRNA is miR-486, which was initially identified as a downstream target of myocardin-related transcription factor-A in cardiomyocytes [12]. Most recently, miR-486 is to be highly induced during myoblast differentiation, and to exert myogenic function.

Besides muscle-specific miRNAs, muscle non-specific miRNAs also play a positive role in regulating the growth and development of skeletal muscle. MiR-24 can induce cardiac hypertrophy in vitro. In adult terminally differentiated cardiac muscle and skeletal muscle cells, the expression of miR-24 is at the basal level, while it is overexpressed at the initial stage of cardiac muscle differentiation [27]. Up till now, the specific function of miR-24 is still unknown [28]. The transforming growth factor β/mothers against decapentaplegic homolog 3 (TGF-β/Smad3) signal pathway can result in the lower expression of miR-24 and restrain myoblast differentiation [29]. The expression of miR-181 is obviously improved during the differentiation process of mouse myoblast cell C2C12, indicating the possibility of miR-181 in regulating myoblast differentiation [28]. Further research has found that miR-181 promotes myoblast differentiation by reducing the expression of its target gene *Hox-AII* (a myoblast differentiation suppressor) [30,31]. MiR-27b, via combining with 3′-UTR in its target gene *PAX3*, can suppress *PAX3* so as to ensure the normal procedure of myoblast differentiation [30]. When miR-27b expression is restrained and *PAX3* expression remains at a certain level, cell proliferation will be promoted and its differentiation will be delayed. During the transitional period from cell proliferation to differentiation, some miRNAs are up-regulated, while some others are down-regulated [30]. The enhanced expression of P27, as a suppressor of cell cycle and a common target of miR-221 and miR-222, is highly correlated with the reduction of miR-221 and miR-222 [32]. MiR-148a is induced during the differentiation of myoblasts, which can down-regulate the expression of Rho-associated protein kinase 1 (ROCKl) to promote cell differentiation [33]. MiR-125b also can negatively regulate the skeletal muscle differentiation process by targeting insulin-like growth factor 2 (IGF-II) [34]. MiR-23a inhibits myocyte differentiation by inhibiting the expression of heavy chain of fast muscle actin [35]. MiR-199a-3p is highly expressed in skeletal muscle and can regulate a number of genes in the IGF1/Akt/mTOR signaling pathway to regulate the differentiation of C2C12 [36]. MiR-186 also can inhibit the differentiation of myoblasts by inhibiting myogenin regulation [37].

In summary, miRNAs are widely present in skeletal muscle, and play an irreplaceable adjustment function in skeletal muscle cell proliferation, differentiation, apoptosis, development and other physiological processes. Therefore, miRNA gene transfected expression or gene therapy such as miRNA interference may become an effective biological pathway for the treatment of a series of muscle diseases in the future.

## 4. MiRNA and Muscle Atrophy

Muscle atrophy, with the typical symptom of muscle quality loss, results from the increased protein degradation or the reduced protein synthesis in skeletal muscle [38]. Based on different pathogenesis, muscle atrophy can be divided into the primary or secondary disorders of skeletal muscle, and aging-driven sarcopenia [1]. Primary muscle atrophy is directly caused by muscular disorders such as Duchenne muscular dystrophy (DMD), while secondary muscle atrophy results from diseases and external factors including weightlessness effect. At present, greater attention has been exerted to research on miRNAs and secondary muscle atrophy.

The weightlessness of skeletal muscle caused by spaceflight or hind limb suspension can reduce the size and strength of skeletal muscle, and promote its transformation into glycolytic-type muscle fiber [39]. Compared with mice in normal conditions, 272 miRNAs in the gastrocnemius of mice subjected to 11-day space flight are changed significantly [40]. Among them, miR-206 is reduced remarkably, while miR-1 and miR-133a have a decreasing tendency. In many muscle atrophy models, both *MAFbx* (Atrogin-1), a gene related to muscle atrophy, and myostatin (a suppressor of muscle growth) have increased simultaneously [41]. Up till now, it is still not clear whether miR-206 can directly or indirectly restrain the expression of atrophy-related genes [42]. The research on miRNA expression of skeletal muscle has been conducted in mice to inspect whether muscle atrophy caused by hind limb suspension can change miRNA expression [40]. After suspending mouse hind limbs for seven days, the expression of miR-107, miR-208b, miR-221 and miR-499 in soleus muscle tissue is remarkably reduced, and miR-23b presents a decreasing trend. Unlike the muscle atrophy model that resulted from spaceflight weightlessness, the expression of miR-206 is not reduced in this experiment, owing to different experimental subjects, muscle types and experiment durations [10]. In addition, the disuse muscle atrophy model has been also established by conducting an experiment of seven-day bed rest, and miR-1 and miR-133a in *Vastus lateralis* tissues from a muscle atrophy model have been reduced by approximately 10% through the evaluation of biopsy [43]. Denervation can also result in disuse muscle atrophy [44]. Amyotrophic lateral sclerosis (ALS) is a neurodegenerative disease with the symptoms of motor neuron loss, muscle atrophy and paralysis. MiR-206 reveals the significant increase in ALS mouse model probably due to its function to correct muscle innervation [45]. This result indicates that improving miR-206 expression is likely to be the correctional treatment of innervation in the future. Coincidently, exogenous miR-206 can promote the compensatory regeneration of neuromuscular synapse, and slow down ALS course. Partial effects of miR-206 illustrated above are realized through the signal transduction pathway of HDAC4 and fibroblast growth factor [45].

Sarcopenia begins at 40 years old, and becomes more and more serious with the increase of age. Although the existence of sarcopenia has been specified, its generation mechanism is not clear. With the in-depth understanding to miRNA, its role in aging is more and more obvious. Skeletal muscle biopsy has shown that pri-miRNA-1-1, pri-miRNA-1-2, pri-miRNA-133a-1 and pri-miRNA-133a-2 in muscle tissue of old people (68–72 years old) are overexpressed when compared with young people (27–31 years old), while no change for pri-miR-206. On the other hand, the expression of miR-1 and miR-133a in muscle tissue of both young population and old population is same [46]. It is not clear why aging can cause the change in miRNA expression and what kind of impact on such change will have. Large samples are needed to conduct further exploration to figure out whether the changes in skeletal muscle associated with the aging process are caused by abnormal functions of miRNAs. In addition, when comparing old population, potential impacts of other factors including physical activity and nutritional status on old population have to be considered, because such factors can also affect miRNA expression in muscle tissues. Moreover, microarray analysis of miRNA expression has been conducted in muscle tissues of 12-month-old and 24-month-old mice to reveal the increased expression of miR-206, miR-698 and miR-468, and the reduced expression of miR-181a, miR-221, miR-382, miR-434 and miR-455 during the aging process [47]. Previous research has confirmed the effect of miR-206 on the hypertrophy of skeletal muscle, so the increased expression of miR-206 in aging is probably to prevent further development of muscle atrophy [48]. Although the specific targets of miR-698 and miR-468 in skeletal muscle have not been confirmed, cardiotrophin 1 has been predicted as the target of miR-698 because cardiotrophin 1 can restrain myogenic regulatory factor during myoblast differentiation [47]. MiR-221 is one of the miRNAs to prevent the aging of skeletal muscle, and its high expression can restrict myoblast differentiation. During the normal differentiation of myoblasts, the reduced expression of miR-221 can be observed [32]. In a word, this miRNA is productive in skeletal muscle to maintain its gene phenotype for the differentiation during the aging period [32]. Research has also shown the participation of miR-455 in the differentiation of brown adipocytes [49], but how low expression of miR-455 regulates the function of aged skeletal muscle needs to be further explored. Recent research on RNA sequencing has indicated the expression of different miRNAs in skeletal muscle of young and old macaques [50]. In the skeletal muscle of old macaques, several new miRNAs including miR-744-5p are overexpressed. Among all myomiRs, miR-181a is down-regulated, and caloric restriction can restore it to the original level. According to previous reports, tumor necrosis factor-α (TNF-α), interlukin (IL)-6, IL-1β and IL-8 are the targets of miR-181a [51], and the reduction of miR-181a probably results in the increase of above inflammatory factors during the aging period. Through comparing and analyzing miRNA expression in muscle tissues of four-month-old and 24-month-old mice, some miRNAs are up-regulated, while some are down-regulated [52]. During the process of sarcopenia or aging, the expression of miR-29 is always increased. The targets of miR-29 are IGF-1, p85a and myeloblastosis-related protein B (B-myb) in muscle tissue of old mice. IGF-1 and p85a are important regulatory proteins for protein translation through miR-29 to restrain the synthesis of myogenin and protein, thereby reducing protein components during sarcopenia and other muscle atrophy [53]. The excessive expression of miR-29 in skeletal muscle can result in the increased expression of some cell-cycle-hindering proteins such as cyclin-dependent kinase inhibitor 2A and phosphorylated retinoblastoma protein (pRB), and such increase can be observed in the skeletal muscle of old people [52]. Therefore, miR-29 directly or indirectly regulates the expression of various proteins, and finally results in the atrophy of skeletal muscle during the aging period.

Recently, the obvious change of several miRNAs with high expression in muscle has been identified and detected in plasma and serum during the process of muscle disorders [54]. Serum miRNAs may come from the active secretion process of histiocytes, while mature miRNAs are coated with lipids or lipoproteins to form exosomes and secrete into blood. The exosomes in blood can enter recipient cells by endocytosis and detach its coating, thereby releasing miRNA to exert biological functions [55]. Previous studies have conducted the comprehensive miRNA expression profiles by miRNA-seq analysis in *Tibialis anterior* muscle and serum from a disuse-induced atrophy mouse model, mimicking acute atrophy following long-term bed rest after comparing with young and old mice [56]. Also, Mizuno has determined the expression levels of specific serum circulating miRNAs to explore the possibility as new biomarkers for muscular diseases, whose results have confirmed that several muscle-specific miRNAs such as miR-1, miR-133a and miR-206 in serum can be useful and reliable biomarkers for muscular dystrophy [57]. Similarly, miR-1, miR-133, and miR-206 have been evaluated as the new and valuable serum biomarkers for the diagnosis of DMD and the monitoring of therapeutic intervention outcomes in humans [58]. Hence, serum miRNA expression level or change can reflect physiological or pathological changes and has a great potential as a biomarker for diagnosing, monitoring disease progression, or evaluating therapeutic efficacy of a series of muscle diseases.

Muscle atrophy is characterized by reduced mass and weak strength of skeletal muscle due to either accelerated degradation or slow synthesis of the protein [38]. Several signal pathways have confirmed to be involved in the regulation for the mass or fiber size of skeletal muscle. Among various signal pathways, IGF-1 and myostatin signal pathways have gained tremendous attention. It has been found that IGF-1 plays a positive role in myogenesis, and the activation of myostatin can hinder muscle growth and even result in muscle atrophy [59]. After combining with its tyrosine kinase (TK) receptor IGF-1R, IGF-1 activates phosphatidylinositol-3-kinase (PI3K) through insulin receptor substrate 1 (IRS1). Sequentially, activated PI3K produces phosphatidylinositol-3,4,5-triphosphates (PIP3) and induces the activation of Akt, thus correspondingly activating mTOR in mammals and regulating ribosomal protein S6 kinase (p70S6K) (a kinase controlling downstream protein synthesis) and 4E-BP1 [60]. In addition, Akt can inactivate glycogen synthase kinase 3β (GSK-3β) to prevent protein translation [61], and restrain the nucleus translocation of Forkhead box O (FoxO) transcription factor family, because FoxO is the key in regulating muscle ring-finger-1 (MuRF1) and Atrogin-1 associated with muscle atrophy, and its inactivation can restrain muscle protein degradation [62]. According to the above descriptions, the IGF-1/Akt signal pathway plays an important regulatory role in the hypertrophy of skeletal muscle. The muscle-specific miR-1 can regulate the growth and differentiation of myoblasts through its target IGF-1. Previous study has also found that during the differentiation of myoblast cell clone C2C12, and the protein expression levels of miR-1 and IGF-1 are negatively correlated, because IGF-1 inactivates the transcription factor FoxO3a to reduce miR-1 expression [63]. In addition, after the transfection of miR-1 and miR-133a into the myocardium in neonatal mice, the IGF-1-induced cardiac hypertrophy is hindered [64]. Based on a study on another muscle-specific miR-206, conclusion has been made that miR-206, through 3′-UTR in its target IGF-1, directly regulates IGF-1 at the level of mRNA, and the reduced miR-206 can remarkably improve the level of IGF-1 mRNA [65]. However, other investigations have discovered that IGF-1R can be regulated by miR-133 [66]. There is a binding site of miR-133 in the 3′-UTR of IGF-1R, and the increased miR-133 can significantly restrain the post-transcriptional expression of IGF-1R in C2C12 cells. The excessive expression of miR-133 or the knockout of IGF-1R can reduce the phosphorylation of Akt in IGF/PI3K/Akt signal pathway [63,64]. It has been reported that miR-486 can affect the expression and activity of FoxO1 and phosphatase and tensin homolog (PTEN) [12]. Through utilizing the miR-486 analogue, it has been found that miR-486 can hinder the dexamethasone-induced protein degradation without any effect on protein synthesis. MiR-486 reduces the expression of PTEN and increases the phosphorylation of Akt, thus resulting in the declined translation of FoxO1. As for the chronic kidney disease of mice, the increase of miR-486 in muscle tissue can restrain the expression of MAFbx/Atrogin-1 and MuRF1, and enlarge fiber size of skeletal muscle [67]. The miR-486 can also restrict Pax7 to promote satellite cell differentiation [68]. Such researches have shown that miR-486 plays a positive role in hindering muscle atrophy from various aspects. Therefore, it is probably a potential therapeutic agent for muscle atrophy. In addition, based on the results of current research, miR-128a is the only miRNA to enhance the quality of skeletal muscle [69]. The high expression of miR-128a is observed in brain and skeletal muscle, and its overexpression is also observed during myoblast differentiation [69]. Because IRS-1 has been a specific target of miR-128a [70], and the excessive expression of miR-128a can restrain myoblast proliferation [24], and reduce the protein content of IRS-1 and Akt activity. The in vitro experiment has found that restricting miR-128a can increase C2C12 myotube size and improve protein content of IRS-1 and Akt activity [69]. It is worthwhile to note that after mice are administered with miR-128a antagonists or inhibitors for four weeks, the size of their skeletal muscle reveals an obvious increase [71]. MiR-128a can restrict glioma growth through another target p70S6K, so probably it can negatively regulate the anabolic route of IGF-1/Akt [72].

Myostatin is the superfamily member of TGF-β. Different from the regulatory effect of IGF-1/Akt, it restrains the improvement of skeletal muscle quality. The myostatin signal is transmitted by receptor serine/threonine kinases (RSTKs) including activin receptor type IIB (ActRIIB) and activin receptor-like kinases (ALK4/ALK5) [73]. After myostatin combining with its receptor, ALK4/ALK5 activates myostatin by phosphorylated C-terminal domain of Smad2 and Smad3 [73]. A compound is formed by phosphorylated Smad2/3 and Smad4, and then transferred to the nucleus for the transcriptional regulation of the target gene. According to previous research, satellite cells have always regarded as the major cause of myostatin-deficiency-induced muscle hypertrophy, because myostatin can restrain the activation and self-renewing of satellite cells [74]. However, several research groups afterwards have discovered the direct impact of myostatin on the synthesis of myofibrillar proteins [75]. Recent research has shown that satellite cells play little role in myostatin-deficiency-induced muscle hypertrophy [76,77].

More and more studies have shown that miRNA is involved in the regulation of myostatin signal pathway. According to recent reports, miR-27a can promote myoblast proliferation by directly restraining myostatin [78]. 3′-UTR in myostatin has the assumed recognition sequence of miR-27a and miR-27b, and the sequence is highly homologous among widespread species across vertebrates. Another research has demonstrated that miR-27b can vigorously reduce the activity of 3′-UTR-luciferase in myostatin [79]. After the sudden change of miR-27b recognition sequence, the activity of myostatin 3′-UTR in C2C12 myotube reveals the increase by nearly two times, and the degradation of myostatin mRNA exhibits reduction at the same time [79]. TNF-related weak inducer of apoptosis (TWEAK) can cause muscle atrophy by reducing miR-27a and miR-27b. After the knockout of myostatin gene in mice, muscle-specific miR-1, miR-133 and miR-206 are greatly up-regulated, which could result in the enhanced expression levels of MEF2 of Myf5 [80].

## 5. Effect of Exercise on miRNA

Skeletal muscle has great contractility, and its contractile activity plays a vital role in maintaining muscle size [81]. Exercise can serve as a powerful stimulus to activate satellite cells and propel protein synthesis in muscle; especially resistance exercise can promote muscle fiber hypertrophy and improve skeletal muscle quality [82]. Previous research has shown that exercise can result in various patterns of gene expression and activate signal pathways [83]. The change in gene expression is partly due to the change of miRNA expression that can strengthen exercise adaptation through specific target genes and its signal pathways [84]. 

**Figure 1:**
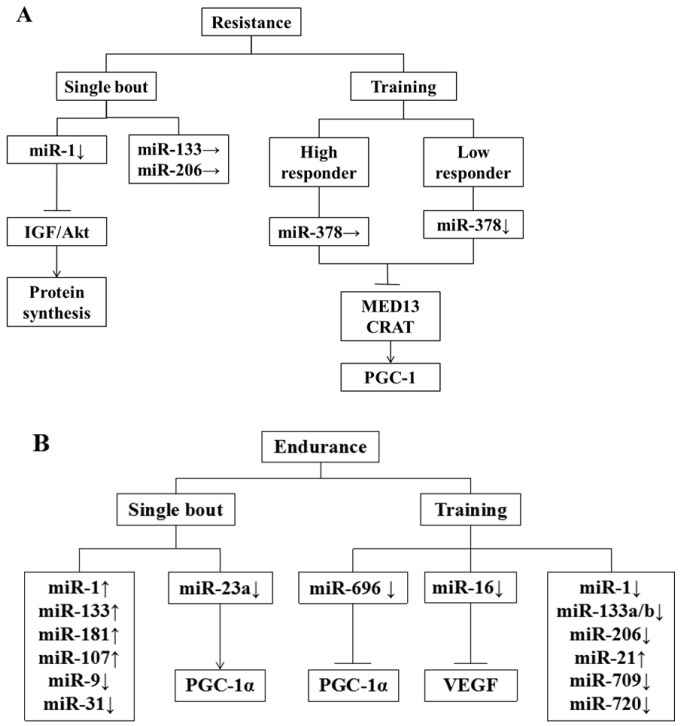
Effect of exercise on miRNAs. (**A**) A single bout of resistance exercise in men reduces miR-1 expression and does not affect the expression of miR-133a and -206. The miR-1 can promote protein synthesis through the IGF-1/Akt signal pathway. The change in the expression of miR-378 is positively correlated with the improved quality of skeletal muscle due to resistance exercise. Similarly, miR-378 inhibits PGC-1β-mediated mitochondrial metabolic effects by MED13 and CRAT; (**B**) a single bout of endurance exercise in mice reduces the expression of miR-23a and increases the expression of miR-1, -181 and -107. Endurance training increases the expression of miR-21 and decreases the expression of miR-696, -709 and -720 in mice. ↑ increased; ↓ decreased; → unchanged; ⊥ inhibited. IGF: insulin-like growth factor; Akt: protein kinase B; PGC-1: peroxisome proliferation-activated receptor-γ coactivator-1; MED13: mediator complex subunit 13; CRAT: carnitine acetyltransferase; VEGF: vascular endothelial growth factor.

Resistance exercise, usually with high strength and short duration, can improve anabolism and promote the synthesis of contractile protein and structural protein in muscle so as to increase muscle size [85]. After taking six-hour resistance exercise with the load of 70% 1RM, both young and old subjects have witnessed a declined expression of pri-miR-1-2 and pri-miR-133a-1 through the biopsy of their muscle tissues, while their pri-miR-206s are up-regulated after exercise for 3 and 6 h [86]. Among all mature miRNAs, only miR-1 is up-regulated, while there is no change for the expression of miR-133a and miR-206 after taking exercise for 3 and 6 h [86]. Through IGF-1/Akt signal pathway, the expression of miR-1 can promote protein synthesis and hypertrophy of skeletal muscle [87]. After taking 12-week resistance exercise, 56 adult subjects are divided into high-response team and low-response team based on their changes of lean body weight (LBW). The detection of 21 rich miRNAs in their muscle tissues has shown that the expression of miR-378, miR-29a and miR-26a in the low-response team is declined, while in the high-response team is regarded as the team enjoying the compensatory effect of exercise, and there is no change for the expression of such three miRNAs. It has been inferred that the change in the expression of miR-378 is positively correlated with the improved quality of skeletal muscle due to resistance exercise, and the stable expression of miR-378 is probably in connection with maintaining LBW [88]. The in vitro experiment afterwards has confirmed the above speculation, and found that the inhibitory factor MyoR, the target gene of miR-378, plays a critical role in promoting myoblast differentiation [89]. During the process of endurance exercise, miR-378 can inhibit peroxisome proliferation-activated receptor-γ coactivator-1beta (PGC-1β)-mediated mitochondrial metabolic effects under the action of mediator complex subunit 13 (MED13) and carnitine acetyltransferase (CRAT) [89], as summarized in Figure 1.

Endurance exercise can increase mitochondrial size and blood capillary density, and improve the oxidation of carbohydrates and lipids [90]. After taking endurance exercise, the expressions of miR-1 and miR-133a in muscle tissue from young people are improved [91], and the myomiR changes resulted from endurance exercise are related to the increased expression of myogenic transcriptional regulatory factors such as myogenic differentiation 1 (MyoD1), myogenin and MRF4 [92]. The expression of miR-1, miR-133a/b and miR-181a in muscle tissues of the mice after taking 3 h endurance exercise is improved, while the expression of miR-9, miR-23a/b and miR-31 is declined [93]. When mice are exhausted after taking 90 min endurance exercise on the treadmill, their miR-1, miR-181 and miR-107 that are closely related to the differentiation and development of myoblasts are up-regulated, while their miR-23 related to the increase of peroxisome proliferation-activated receptor-γ coactivator-1α (PGC-1α) is down-regulated [94]. After taking four-week running as endurance exercise, miR-21 in gastrocnemius of mice is overexpressed, while miR-696, miR-709, and miR-720 are down-expressed [95]. The decline of miR-696 is negatively correlated with the protein level of PGC-1α [95]. The temporary increase of miR-696 in C2C12 myotube has no effect on the expression level of PGC-1α mRNA, but it can restrain its protein level [95]. On the contrary, after the myotube transfected with miR-696 inhibitor, the protein expression of PGC-1α is increased, while the expression of PGC-1α mRNA does not change [96], suggesting that miR-696 may inhibit the process of mRNA translation to regulate PGC-1α. PGC-1α is an important transcription activator in skeletal muscle, which can regulate the functions of skeletal muscle from various perspectives including mitochondrial biological oxidation, substrate oxidation and muscle fiber type transition, and restrain muscle atrophy according to current studies [97]. This study also indicates that the regulation mechanism of miRNA to PGC-1α may be different during acute and long-term endurance exercise.

After swimming training, the expression of miR-16 is significantly reduced in the rat flounder, accompanying elevated vascular endothelial growth factor (VEGF) and increased capillary density. Subsequently, research confirmed that VEGF is the target of miR-16, and it combines with miR-15a to regulate the angiogenesis. These changes indicate that miRNA expression variation caused by gene adjustment network plays an indispensable role in regulating endurance exercise adaptation.

In the human body, the expression of miR-1 and miR-133a can be significantly improved in the lateral femoral muscle after 60 min bicycle movement, and after 12 weeks of training, miR-1, miR-133a/b and miR-206 reveal a significant decrease. At the same time, endurance motor ability, VO_2max_ and insulin sensitivity are significantly increased, which indicates the effects of prolonged endurance training are contrary to acute endurance exercise. Similarly, miR-1, miR-133, miR-101 and miR-455 are reduced in skeletal muscle after six weeks of pedal bicycle exercise. Thus, the variation of miRNA caused by movement is changed by the environment. Therefore, gain-of-function or loss-of-function animal models are needed to support the causal relationship between miRNA and skeletal muscle movement adaptation.

Endurance exercise also can induce change in the expression of plasma-based circulating miRNAs. MiRNAs secreted into blood can display endocrine function. After 90 days of acute exhaustive exercise, the circulating miR-21, miR-221, miR-146a and miR-222 associated with angiogenesis and inflammation are up-regulated. Interestingly, circulating miR-146a and miR-20a have linear relationship with VO_2max_ [98]. Thus, the circulating miRNAs may be the cardiovascular adaptation biomarkers during exercise. MiR-486 can inhibit FoxO responsible for the degradation of protein, while peripheric miR-486 is decreased both in acute endurance exercise and long-term endurance exercise. The decline of circulating miR-486 is likely to promote protein catabolism caused by intensive endurance training [99]. In addition to endurance training, the influence of acute resistance exercise on circulating miRNAs has also been explored. After three days of resistance training (bench press and leg press) with the intensity of 70% VO_2max_, miR-149 is increased, and miR-146a and miR-221 drop in serum. On the other hand, resistance exercise can significantly improve circulating miR-133 related to muscle development [100]. Different exercise modes can cause the specific adaptations through the regulation of blood miRNAs. Although the detailed mechanism needs further exploration, miRNA is likely to become an indicator for monitoring exercise training status, evaluating exercise capacity, and possibly screening sport talents in the future.

All above research has shown that exercise can regulate miRNA expression, so the studies on which signal pathways can execute miRNA expression during exercise intervention, and how miRNAs regulate exercise adaptation will become very important. The discovery of which miRNA is related to exercise adaptation and which miRNA can imitate exercise-induced change will vigorously promote the development of miRNAs in muscle tissue.

## 6. MiRNA as a Novel Research Direction of Muscle Atrophy

As mentioned above, many miRNAs play a significant role in the regulation of muscle atrophy and the maintenance of muscle mass. Therefore, drug preparation using miRNA as the target may be a new strategy to treat muscle atrophy. During the development process of DMD with overexpressed miR-199a-5p-induced myofibrosis, cell membrane separation, cell edema and damage, and the expression of miR-199a-5p can be regulated by SRF so that the suppression of SRF signaling can reduce the transcription of miR-199a-5p. Therefore, miR-199a-5p is the target for the regulatory factor of regulating proliferation and differentiation [101]. The extensive muscle necrosis and damaged myofibers in *MEF2A* knockout mice can be rescued through the overexpression of miR-410 and miR-433 [102]. MiR-31 inhibits the expression of dystrophin, and interfering miR-31 can effectively regain dystrophin activity [58]. MiR-486 can result in the changed kinetics of the regeneration during muscle fiber cell cycle. Transferring miR-486 into muscle tissue by electroporation can slow down the up-regulation of Atrogin-1 and MuRF1, thus preventing chronic kidney disease (CKD) mouse muscle amyotrophy [67].

## 7. Conclusions and Expectation

In this article, the regulatory roles of miRNAs in muscle regeneration and muscle atrophy have been reviewed, which provides the highlight that miRNAs have an important role in mediating the adaptation of skeletal muscle to various exercise modes and executing tackling of aging-associated atrophy or injury of skeletal muscle. MiRNAs are involved in many biological processes, but their molecular mechanisms are still not clear. The following aspects may be the future hot topics of miRNA studies:

(a) The abnormal regulation of miRNAs can lead to a series of diseases such as DMD, ALS and sarcopenia. Whether miRNAs can be used as the new therapeutic targets or agents to detect and treat these diseases is highly required to be explored.
(b) The changes in miRNAs and related proteins are observed in diverse muscle disorders and diseases. Whether miRNAs can be regulated in human body by generic and biochemical means is necessary to be explored, so as to accomplish the prevention and treatment of skeletal muscle injuries or corresponding disorders.
(c) Exercise can modulate the expression of miRNAs for controlling a series of physiological and pathological changes. Exploring exercise-induced miRNAs as the targets will be beneficial for the development of novel and effective nutritional supplements, drugs or other intervention strategies for disease prevention and treatment or health promotion.
